# Addressing the unmet clinical need for low-volume assays in early diagnosis of pancreatic cancer

**DOI:** 10.3389/fgstr.2023.1258998

**Published:** 2023-09-19

**Authors:** Daniel A. Sheik, Kaleb Byers, Mini Thomas, Ummadisetti Chinna Rajesh, Kelli Ifuku, Kimberly Kirkwood, Mohammed Al-Haddad, Charles S. Craik, V. Jo Davisson

**Affiliations:** ^1^ Research and Technology Department, Amplified Sciences, Inc, West Lafayette, IN, United States; ^2^ Department of Surgery, University of California, San Francisco, CA, United States; ^3^ Division of Gastroenterology and Hepatology, Indiana University (IU) School of Medicine, Indianapolis, IN, United States; ^4^ Department of Pharmaceutical Chemistry, University of California, San Francisco, San Francisco, CA, United States; ^5^ Department of Medicinal Chemistry and Molecular Pharmacology, Purdue University College of Pharmacy, West Lafayette, IN, United States

**Keywords:** rule-out, rule-in, dysplasia, early diagnosis, surface-enhanced Raman spectroscopy, pancreatic cancer, pancreatic cystic lesions

## Abstract

The incidental detection of pancreatic cysts, an opportunity for the early detection of pancreatic cancer, is increasing, owing to an aging population and improvements in imaging technology. The classification of pancreatic cystic precursors currently relies on imaging and cyst fluid evaluations, including cytology and protein and genomic analyses. However, there are persistent limitations that obstruct the accuracy and quality of information for clinicians, including the limited volume of the complex, often acellular, and proteinaceous milieu that comprises pancreatic cyst fluid. The constraints of currently available clinical assays lead clinicians to the subjective and inconsistent application of diagnostic tools, which can contribute to unnecessary surgery and missed pancreatic cancers. Herein, we describe the pathway toward pancreatic cyst classification and diagnosis, the volume requirements for several clinically available diagnostic tools, and some analytical and diagnostic limitations for each assay. We then discuss current and future work on novel markers and methods, and how to expand the utility of clinical pancreatic cyst fluid samples. Results of ongoing studies applying SERS as a detection mode suggest that 50 µL of pancreatic cyst fluid is more than sufficient to accurately rule out non-mucinous pancreatic cysts with no malignant potential from further evaluation. This process is expected to leave sufficient fluid to analyze a follow-up, rule-in panel of markers currently in development that can stratify grades of dysplasia in mucinous pancreatic cysts and improve clinical decision-making.

## 1 Introduction

Pancreatic cancer continues to move toward the top of the list of deadliest cancers, with an estimated 5-year survival rate of around 10% (1). The grim outlook for this disease can be directly attributed to the late stage at which an accurate diagnosis is typically achieved. Pancreatic cystic lesions are potential predictors of progression to cancer, and understanding the progression from cyst to cancer provides an avenue for early pancreatic cancer detection. Pancreatic cystic lesions are incidentally detected in 3 million (and growing) patients annually in the USA and present a real-world clinical problem for diagnosing early-stage malignancy accurately (2). Several studies have estimated from *post mortem* autopsy analysis that 24% of people over 60 years old could pass away with an unidentified pancreatic cyst of variable clinical signficance (3–5). An impediment to early-stage detection is the lack of noticeable symptoms during progression from non-malignant pancreatic cyst to invasive pancreatic cancer until it is too late for meaningful intervention (6–8). At the time of this publication, there are no approved screening methods to detect pancreatic cysts or cancer for the population at large. Thus, a significant problem in the management of pancreatic cystic lesions is simply finding patients in whom a potentially malignant pancreatic cyst has developed and identifying those patients for whom surgical resection is necessary. Conversely, patients with indolent pancreatic cysts that will never progress to malignancy should also be identified to avoid unnecessary surgery. As a result, a significant number of people remain undiagnosed or untreated at a time when surgical resection would be beneficial.

Over the past decade, efforts have focused on improving diagnostic resolution in pancreatic cancer. Novel diagnostic tools, markers, and analytical methods are providing more information about effective approaches to diagnosis. Still, there remains an unmet need for such tools to be translated into clinical settings. This work will discuss the current pathway to diagnosis, including the limitations of clinical diagnostic tools for pancreatic cancer, followed by advances being made in laboratories for translation into clinical settings. This article is not intended to be a comprehensive analysis of every marker and tool reported. Instead, the intention is to identify unmet needs associated with the accurate clinical diagnosis of pancreatic cysts, focusing on the current standards of care, and examine select projects that are seeking translation to the clinic to address these unmet needs and enhance the resolution for this troublesome diagnosis.

## 2 Current pathway to diagnosis

Pancreatic cyst patient management is shaped by three main guidelines (ACG, AGA, and Fukuoka), which seek to incorporate imaging and cyst fluid analyses to guide the management of a pancreatic cyst for surveillance or surgical resection (9–11). Most patients with pancreatic cysts have “indeterminate” cysts in which both the type (mucinous vs. non-mucinous) and histologic grade are radiographically uncertain (12, 13). These patients are often referred for endoscopic ultrasound with fine needle aspiration (EUS-FNA) to extract pancreatic cyst fluid samples for chemical and genomic analyses. Although EUS-FNA is a complex and invasive process, it is preferred to endoscopic solid tissue biopsy with microforceps because of the elevated risks associated with the tissue biopsy procedure and an estimated diagnostic improvement of only 10% (14, 15). As discussed throughout this article, pancreatic cyst fluid is a commonly acellular, highly proteinaceous, and viscous biofluid that contains a wealth of bioinformation and substances that could potentially interfere with clinical assays.

**Figure 1:**
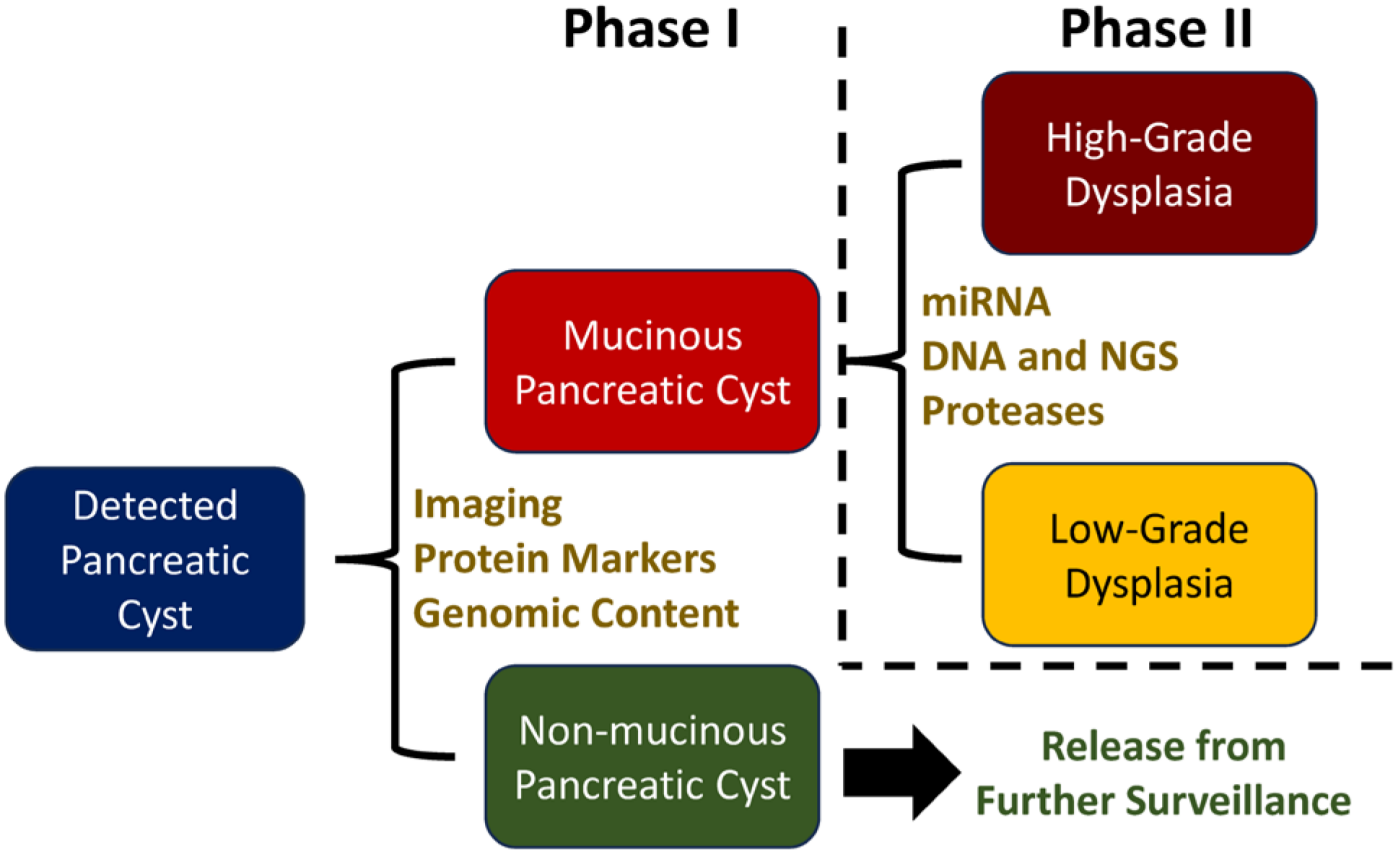
General pancreatic cyst diagnosis strategy and example tests for each phase.

Ideally, the first step in diagnosis is to rule out potential malignancy by differentiating between non-mucinous pancreatic cysts that are predominantly benign from mucinous cysts that have a 10%–50% chance of progressing to cancer (16). With these two cohorts identified, different treatment plans can be crafted, wherein patients in need of further testing of a potentially malignant mucinous pancreatic cyst are prioritized. In this situation, the roughly 20%–40% of patients that undergo an EUS-FNA procedure and are confirmed to have a non-mucinous cyst are spared from further surveillance and the attendant emotional, physical, and financial burdens of repeat doctor visits, diagnostic testing, and monitoring (16, 17). Next, clinicians strive to stratify the mucinous population based on the actual risk of progression to cancer (the rule-in phase). This second diagnosis phase is decidedly more challenging, as the established markers for the rule-out diagnosis have not proven to be adequate predictors of progression to cancer for rule-in determinations (18). As a result, although new marker discoveries are ongoing, there is not currently an accepted method by which pancreatic cysts with high-grade dysplasia or early invasive cancer can be quickly and accurately identified. It is also important to note that this process is rarely as straightforward as presented here or in **Figure 1**. However, multiple technologies have been developed, and continue to be improved, that can accurately determine the type of pancreatic cyst present.

## 3 Current clinical diagnostic tools

### 3.1 Imaging and cytological analysis

Pancreatic cysts are commonly found incidentally during routine imaging for unrelated disorders. Advances in CT and MRI imaging have improved resolution such that more and smaller pancreatic cysts can now be identified, providing an opportunity for earlier diagnosis (19). However, this comes with the drawback that smaller cysts are more challenging to classify and produce less cyst fluid for biochemical analyses. The primary utility of imaging for classification relies on shape/size and location. It has been established over the years that cysts that form within the main pancreatic duct and those that have grown beyond 3 cm are more likely to progress to cancerous lesions than cysts that form in other regions of the pancreas or smaller cysts, however the risk remains highly variable (10). Although helpful, as with any image analysis, this approach comes with a subjective bias that hinders accurate diagnosis. Efforts to avoid image interpretation errors have focused on using machine learning and artificial intelligence methods (ML-AI) to process all available images and more accurately determine the classification of a pancreatic cyst. This work is mainly in the nascent stage, and it shows greater promise in organs like the lung in which the tumor signal-to-background ratio is high. In contrast, the solid components of pancreatic tumors are frequently camouflaged, making them difficult to detect (20). For patients with indeterminate cystic lesions of sufficient size, the next logical step is often to sample the cyst fluid and conduct (bio)chemical analyses.

Use of the EUS-FNA procedure has become more widespread, as the information contained within pancreatic cyst fluid has proven valuable (21–23). This section, and the following sections, will focus on EUS-FNA fluid analysis. In the case of pancreatic cyst fluid, microscopic examination of fluid contents (cytology) has the potential to accurately diagnose a pancreatic cyst with high-grade dysplasia or adenocarcinoma. However, the examination is often non-diagnostic because of the paucity of tumor cells in the fluid, with an estimated 31% success rate for obtaining cells for cytological analysis (24). Furthermore, the sample volume required for slide preparation may exceed 500 µL, which is often unavailable. Although this is a common clinical practice for diagnosing pancreatic cysts, the limited cellularity of pancreatic cyst fluid yields relatively low clinical utility; thus, we will omit any further discussion of cytology.

### 3.2 Cancer protein biomarkers

Biomarkers indicative of cancer have been studied, published, and established in the clinic as screening and diagnostic tools. Every cancer is different, but several biomarkers consistently present themselves during clinical investigations (25, 26). Their ubiquity makes them generally poor predictors of cancer type, but they unquestionably retain utility as indicators of cancer or progression toward cancer when the possibility of cancer arises. In the case of pancreatic cancer, carcinoembryonic antigen (CEA) and amylase have been used extensively in the clinic to support rule-out diagnoses. However, neither marker is individually indicative of either non-mucinous or mucinous pancreatic cyst fluid, with low sensitivity and/or specificity (27, 28). Other markers that suffer similar fates in clinical settings include CA19-9 and glucose; however, glucose has maintained clinical relevance for determining mucinous pancreatic cysts, with a sensitivity and specificity over 80% (29–32). For clinical labs, however, any of these markers represent a seemingly sensible method, as they are all amenable to automated processing on clinical analyzers, with little training or upgrades needed. Importantly, this reliance on convenience is still rife, causing clinical problems for diagnosis.

Clinical analyzers and their associated assays have been developed to work with highly proteinaceous fluids such as blood, serum, or plasma, and some are compatible with alternative biofluids such as urine or saliva. None of these fluids compare with pancreatic cyst fluid’s relatively high complexity and inter-sample variability. A basic review of published investigations into proteomic analyses of pancreatic cyst fluid reveals thousands of individual biomaterials present in any given sample (18, 33, 34). Still more problematic is that high concentrations of biomaterials have been established to cause inaccuracy and/or decreased reproducibility/repeatability in standard assay formats (35–37). For example, viscous materials like mucins present challenges in basic liquid handling, leading to erroneous sample volume transfers and assay results. Furthermore, the sample volume requirements for clinical analyzers often preclude the replicate analysis of multiple markers in single pancreatic cyst fluid samples. The balance at hospital clinics between high-throughput analysis and sample preservation is certainly skewed toward high throughput. Furthermore, the standards set by the analysis of readily available blood samples are often inappropriate for pancreatic cyst fluid samples. Although assays for markers of interest could be run at lower volumes, the analyzers employed in the clinic often require 200–500 µL of sample per run. When these factors are taken together, it is unsurprising that assays of biomarkers that lack sensitivity and specificity, and inhibit multiple-marker analyses due to sample consumption, inevitably lack the capacity for accurate diagnosis.

However, there are alternative protein markers still undergoing translational research that can provide useful diagnostic information. For instance, vascular endothelial growth factor (VEGF) has been shown to be a reliable marker of notoriously difficult to diagnose serous cystic neoplasms (SCNs) (38–40). SCNs are non-mucinous pancreatic cysts that are difficult to distinguish from potentially malignant intraductal papillary mucinous neoplasms (IPMNs) and mucinous cystic neoplasms (MCNs) using imaging or the previously described protein markers (41). Although they constitute an estimated 1%–2% of all pancreatic cysts, avoiding unnecessary surgical intervention for SCNs will improve the quality of life for patients (42). In addition to VEGF, the monoclonal antibody Das-1, originally used as a marker of colonic epithelial phenotype, has shown utility in predicting malignancy in conditions of the upper GI tract (43, 44). Importantly, Das-1 has displayed the unique ability to stratify low- and high-grade dysplasia and invasive cancer in mucinous pancreatic cyst fluid.

### 3.3 Genomic markers and methods of interest

Research into the genetic drivers of pancreatic cancer has found long lists of markers in DNA, RNA, miRNA, and mRNA, and has recently made the logical step toward combination marker panels investigating several genetic components at once. Again, most of the tools have not translated into clinical utility, except for DNA markers that can be amplified and quantified in high-throughput platforms (45, 46). Next-generation sequencing (NGS) is a technique that emerged on the market in the mid-2000s, and has provided a wealth of information for researchers in biology and biochemistry. Every clinical sample prepared for extraction is subject to volume requirements to meet the demand of the extraction method. Although sample preparation for NGS can be conducted with lower volumes, to counter low-genomic-content samples, general estimates for required sample volume are around 500 µL (47). This means that NGS is expected to consume significant resources from a limited pancreatic cyst fluid sample, further constraining the utility of other diagnostic tools.

Regardless, although the fluid is typically acellular, NGS has produced rich genetic information from pancreatic cyst fluid samples. Studies investigating genetic markers or defects have found that NGS can accurately diagnose specific cyst types. A prime recent example of this is the study by Paniccia et al., wherein they investigated 1,933 pancreatic cyst fluid samples for genomic content and were able to diagnose those cysts with negative predictive values (NPVs) and positive predictive values (PPVs) greater than 90% in most cases (48). They also made use of imaging where available to supplement NGS data and further improve the NPV and PPV in select cases. With the cyst type identified, a more practical treatment course can be determined, and this information indeed leads to better diagnostic outcomes for patients. But it is again important to note that cyst type alone does not confer sufficient information about the degree of risk of progression to cancer. Recent efforts have focused on improving analysis algorithms to identify cyst types better and eventually stratify dysplasia.

## 4 Advanced research geared toward clinical translation

### 4.1 Rise of exosomal research and the impact on diagnostics

Recent years have seen a tremendous increase in interest in biological exosomes (49). Broadly speaking, exosomes are vesicles ranging in size from 10—200 nm that are released from cells and contain unique chemical and genetic information. Exosomes are found in many distinct human biofluids, with the majority of research focused on blood-based exosomes, which contain information from any tissue source secreting into the bloodstream. Blood-based exosomes are ideal targets for the analysis of most disease states, including, if not especially, cancer (50). It has been hypothesized that cells use exosomes to inform their local community of different signals and activation patterns (51). Until recently, however, efficient isolation of such material from biological sources was complicated at best, and near impossible at worst.

Multiple methods, including ultracentrifugation, size exclusion chromatography, and immunoaffinity have been used to isolate exosomes with varying degrees of success and recovery. However, a recent study by Hinestrosa et al. demonstrated a clever method for isolating exosomes from plasma, wherein they used an electrode array to attract the charged membrane of exosomes to a solid support from which they can be washed and resuspended for analysis (52). This study investigated exosomes to identify pancreatic, ovarian, and bladder cancers, and analyzed the resulting samples with commercially available protein quantification kits. The compiled data were used to train an algorithm for multiple markers that could accurately detect early-stage cancer, and displayed a high sensitivity for pancreatic cancer. These results suggest that exosomes should continue to be a source of diagnostic information, and it is likely that with some further marker optimization and refined assay development this isolation technology has the potential for widespread adoption in cancer diagnostics.

### 4.2 Protease activity as a functional biomarker

An underutilized approach in the search for clinically translatable diagnostic assays in pancreatic cancer has been the analysis of enzyme activity, as opposed to enzyme mass (53–56). Immunoassays dominate the landscape of biomarker analysis and have been readily adapted into automated systems that improve throughput, reproducibility, and sample handling. ELISA-based methods have provided an incomparable wealth of data about protein expression and are now a mainstay in research and clinical laboratories. However, immunoassays quantify the amount of protein present, which can be ambiguous when investigating enzyme markers as active (or activatable) enzymes are responsible for signaling cascades, metabolism, and phenotypical responses (57–59). There have been publications over the years examining protease cleavage, enzymatic redox reactions, and other activity measurements. Yet still, these assays have struggled to translate meaningfully to clinical settings in the same way that immunoassays have over the decades. One possible reason is that, although protease cleavages are an amplification process, proteolytic activity on substrates is not necessarily as efficient or rapid a process as the commonly used secondary reactions in ELISA formats. Due to the lower amplification of optically active products, it is difficult for traditional optical methods to detect and quantify low activity. It is even more complicated when sample volumes are limited.

Clinical assays rely on optical detection methods including luminescence, fluorescence, and absorbance. New protein biomarkers are routinely identified using mass spectrometry (MS) methods. However, for many of these candidate markers, the expression level is below the limits of detection for traditional optical methods. A path forward is the use of alternative detection technologies such as Raman spectroscopy. Raman spectroscopy is based on an inelastic light scattering event that imbues the scattered photons with chemical and structural information owing to the excitation of molecular vibrational modes. Although only approximately 1 in 100,000,000 photons are predicted to produce Raman scattering, the introduction of a coinage metal (Au, Ag, or Cu) surface has been shown to enhance the Raman scattering intensity by multiple orders of magnitude, leading to ultrasensitive detection limits (60, 61). First discovered in 1974, surface-enhanced Raman spectroscopy (SERS) has become a more practical tool as the cost and size of Raman equipment have dramatically decreased (62, 63). Recent investigators have used SERS to detect single-molecule Raman signals (SMSERS), which provides additional support for the utility of SERS in clinical assays for low-concentration and/or low-volume samples (64–66). Furthermore, the chemical fingerprint information provided by Raman scattering enables discrimination of multiple dye classes (i.e., fluoresceins, rhodamines, cyanines), or dyes with variable isotopic labeling, enabling intrasample multiplexing at incredibly low concentrations (67–70).

The authors of this review have translated assays using Raman-active dyes that have shown detection limits below those of traditional fluorescence measurements (71). Furthermore, Suresh et al. have published the first example of translating a SERS assay for protease activity of gastricsin to a clinically ready platform with a small cohort of human pancreatic cyst fluid samples (72). Pancreatic cyst fluid is a notoriously tricky medium with which to run assays or optically quantify results, and the simplest solution is significant sample dilution to diminish interfering substances. The ultrasensitivity of SERS and a protease activity assay that displayed reduced interference from the sample milieu provided an ideal assay platform for high-dilution quantification. Importantly, the published results were from assays of 1 µL of pancreatic cyst fluid, supporting the analysis of multiple markers indicative of pancreatic cancer from single pancreatic cyst fluid samples without high volume requirements. Currently the group is engaged in a statistically powered study with a larger cohort and a panel of three biomarker assays that is expected to produce an NPV of >95% for identifying non-mucinous pancreatic cyst fluid. Again, all three assays are run using only 12 μL of clinical sample, meaning that these efforts do not disrupt current standards of clinical care, and analyze a sample volume that would be incompatible with contemporary clinical assays.

## 5 Discussion and future directions

Each area of research described in this article has produced undeniable advances in support of decision-making for patients in whom a pancreatic cyst has been detected. However, more work, creativity, and translatable development of new tools are required to produce the desired outcomes for patients. Modifications to assay platforms, or preanalytical steps paired with assays that consume less sample volume, will undeniably expand the utility of limited clinical samples. There are also other approaches seeking to use multimodal analyses to diagnose pancreatic cysts (47, 73). It is undeniable that incorporation of standard clinical data from imaging and cytology into biomarker analyses provides the best diagnostic resolution for patients and clinicians. However, most of the work described here identifies patients who can be ruled out from further consideration. It is the remaining mucinous pancreatic cyst population that requires attention moving forward. Marker identification and translation, combination panels, and collaboration will be vital in addressing the unmet need for dysplasia grading in pancreatic cyst fluid.

## Author contributions

DS: Conceptualization, Writing – original draft, Writing – review & editing. KB: Writing – review & editing. MT: Writing – review & editing. UR: Writing – review & editing. KI: Writing – review & editing. KK: Writing – review & editing. MA: Writing – review & editing. CC: Writing – review & editing. VD: Conceptualization, Writing – review & editing.
